# Social Isolation Patterns and Health Outcomes in Midlife and Later Life

**DOI:** 10.1093/geroni/igab046.142

**Published:** 2021-12-17

**Authors:** Meng Sha Luo

**Affiliations:** Zhejiang University, Hangzhou, Zhejiang, China (People's Republic)

## Abstract

Conceptualizing social isolation as a multidimensional construct encompassing social networks, social contacts, perceived support and loneliness, this research aims to: (1) identify patterns of social isolation trajectory among middle-aged and older adults in the U.S.; (2) investigate how different patterns of social isolation trajectory are related to adults’ physical, mental, cognitive, and overall health. Latent class growth modeling was used to examine social isolation trajectory patterns over nine years in a national sample of 6,457 adults aged 51+. Four patterns of social isolation trajectory were identified: severely isolated, moderately isolated, subjectively integrated, and objectively integrated. The objectively integrated group reported the best physical, mental, cognitive, and overall health, whereas the severely isolated group reported the worst. The moderately isolated and subjectively integrated groups fell in between, with the latter displaying relatively better health outcomes. Findings support a close relationship between poor health and long-term social isolation.

